# Anal incontinence and quality of life following operative treatment of simple cryptoglandular fistula-in-ano: a prospective study

**DOI:** 10.1186/s13104-017-2895-z

**Published:** 2017-11-07

**Authors:** Umesh Jayarajah, Dakshitha Praneeth Wickramasinghe, Dharmabandhu Nandadeva Samarasekera

**Affiliations:** Department of Surgery, Faculty of Medicine, University of Colombo, Kynsey Road, P. O. Box 271, Colombo 8, Sri Lanka

**Keywords:** Fistula-in-ano, Quality of life, Anal incontinence

## Abstract

**Background:**

Anal incontinence is a known complication following operative treatment of fistula-in-ano which can significantly impact the quality of life. This study was aimed to objectively assess the impact of operative treatment of simple fistula-in-ano on quality of life related to anal incontinence. Therefore, a prospective study was conducted in 34 patients who underwent surgery for fistula-in-ano over a period of 24 months. Quality of life and incontinence were assessed using fecal incontinence quality of life (FIQL) scale and Cleveland clinic incontinence score (CCIS) preoperatively and after a minimum of 12 months follow up (mean-27 months, range 12–40 months). The difference in FIQL and CCIS was analysed using Wilcoxon Rank test and Mann–Whitney U test.

**Results:**

The median age of the participants was 42.5 years (range 22–63, males = 30). The majority had a trans-sphincteric tract (n = 22, 65%). Superficial tracts and inter-sphincteric tracts were found in 8 (24%) and 4 patients (12%). The overall preoperative and postoperative rates of incontinence were 18 and 38% respectively, but the severity was low. The mean overall FIQL was 16.0 (SD ± 0.4) preoperatively and 16.1 (SD ± 0.4) postoperatively. Considerable difference was seen in the scale measuring “depression/self-perception” (p = 0.012). Only 1 patient (3%) had reduction in scale “lifestyle” which measures the impact of incontinence on day-to-day activities.

**Conclusions:**

Analysis of a cohort of simple cryptoglandular fistula-in-ano with low pre-operative incontinence showed no worsening in the FIQL following successful treatment despite minor worsening of incontinence. Since greater improvement was noted in scale measuring depression/self-perception, psychological interventions may be helpful before surgery to improve quality of life.

## Background

The operative treatment of fistula-in-ano remains a challenge as it is essential to achieve a cure while minimizing postoperative complications. The most important factors that determine outcome are recurrence and anal incontinence following surgery [1]. Anal incontinence is a complication that can significantly affect the quality of life of the patients [2]. The reported overall rates of incontinence vary up to 40% depending on the type of fistula and the operative treatment modality used. However, the majority of patients had minor incontinence following surgery [3]. Studies have shown that simple fistulae also carry a risk of incontinence though not as high as following surgery for complex fistulae [4]. Furthermore, studies have also shown that quality of life and patient satisfaction may be low because of anal incontinence despite a complete cure [1].

There has been a few previous studies examining QOL in anal fistula using the gastrointestinal quality of life index (GIQLI) [5]. The GIQLI was developed from patients with pathologies in the upper gastrointestinal tract, such as peptic ulceration and biliary disease, and it assesses symptoms such as chronic abdominal pain, reflux symptoms, eating habits and bowel habits which are usually not affected in peri-anal fistulae unless it is associated with a known aetiology such as inflammatory bowel disease or TB. Therefore, it is not an accurate assessment for cryptoglandular anal fistula [6].

Studies on quality of life related to anal incontinence following surgery are limited and the authors could not find any prospective studies which had compared preoperative and postoperative quality of life related to anal incontinence. Therefore, this study was aimed to objectively assess the impact of operative treatment on anal incontinence quality of life in simple cryptoglandular fistula-in-ano.

## Methods

A prospective analysis was done. All patients who underwent successful operative treatment for fistula-in-ano from 2012 January to 2013 December at the Professorial Surgical Unit at the National Hospital of Sri Lanka were included in this study. Patients with other comorbidities or psychological conditions that may affect quality of life were excluded. Sample size was determined using the results of 2 previous studies which assessed incontinence following fistula surgery [6, 7], targeting a 95% confidence interval and 80% power to detect worsening of incontinence. This yielded a minimum sample size of 33.

All surgeries were done by a single consultant colorectal surgeon. Those who had fistulae with multiple external openings, high trans-sphincteric fistulae, supra-sphincteric fistulae, extra-sphincteric fistulae, or had high blind extensions or horseshoe tracts or were anterior in a female patient were excluded as they were complex fistulae [8, 9]. A biopsy was done in all patients to exclude any primary cause.

The instrument used to assess the quality of life related to anal incontinence was fecal incontinence quality of life (FIQL) scale which is a validated and a widely accepted tool [10]. It consists of a total of 29 items which form four sub scales. They are lifestyle (10 items), coping behavior (9 items), depression/self-perception (7 items) and embarrassment (3 items). Psychometric evaluation of these scales demonstrated that they are both reliable and valid. The sub scales have satisfactory test/retest reliability and acceptable internal reliability (Cronbach’s alpha > 0.70) [10].

The degree of incontinence was measured objectively using the Cleveland clinic incontinence score (CCIS) which is a widely accepted validated score to measure anal incontinence [11]. It consists of five questions to assess the degree of incontinence (solid, liquid, gas, wears pad, lifestyle alteration). The frequency of each type of incontinence is rated on a scale ranging from 0 (never) to 4 (always or to once a day) so that the sum of the frequencies add up to a total score that may range from 0 to 20. Higher scores indicate higher levels of incontinence.

These tools were administered as an interviewer administered questionnaire to minimize discrepancies. All patients gave informed written consent to be included in the study. Ethical clearance was obtained from the Ethical Review Committee of the National Hospital of Sri Lanka.

Each person’s FIQL and CCIS were assessed at two points, preoperatively and after a minimum follow up period of 12 months after the last surgery. A follow up period of 12 months was chosen to allow adequate time for complete healing and to a certain extent, exclude recurrence. After the follow up period of 12 months, patients were assessed by a clinical examination to confirm the absence of recurrence.

Data were analysed using SPSS 17.0 statistical software (SPSS Inc., USA). Continuous variables were expressed using means ± standard deviations. Wilcoxon Rank test was used to analyse whether there is a statistical difference between preoperative and postoperative scores and Mann–Whitney U test was used to determine associations. All statistical testing was performed at the 0.05 significance level.

## Results

Thirty-four patients participated in this study. The median age of the study participants was 42.5 years (range 22–63). The majority of patients were males (n = 30, 88%). The median number of surgeries that the patients had undergone was 2 (range 1–6). The patients were followed up for a mean duration of 27.47 months (range 12–40). The Parks classification [9] was used to classify all fistulae at the time of surgery. The majority had a trans-sphincteric tract (n = 22, 65%). Superficial tracts were found in 8 patients (24%) and inter-sphincteric tracts were found in 4 patients (12%).

**Fig. 1:**
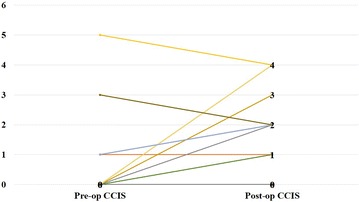
Figure plotting pre and post-op CCIS scores of individual patients

The mean preoperative CCIS was 0.4 ± 1.1 and after follow up the CCIS was 0.9 ± 1.3 out of a maximum score of 20. The difference seen was statistically significant (p = 0.039). The greatest significance was in incontinence to flatus (p = 0.013). However, no patients had significant soiling for solid faeces (Table 1). Plot showing pre and post-operative CCIS scores of individual patients is shown in Fig. 1.

**Table 1:** Comparison of mean preoperative and postoperative incontinence score

	Pre-op CCIS ± SD	Standard error	Post-op CCIS ± SD	Standard error	p value
Total CCIS score	0.4 ± 1.1	0.19	0.9 ± 1.3	0.03	*0.039*
Incontinence to solids	0.06 ± 0.2	0.04	0.03 ± 0.2	0.03	0.564
Incontinence to liquids	0.06 ± 0.2	0.04	0.06 ± 0.3	0.06	–
Incontinence to flatus	0.26 ± 0.8	0.13	0.74 ± 1.3	0.22	*0.013*
Use of pads for stools	0.0 ± 0.0	0.0	0.0 ± 0.0	0.0	–
Lifestyle restriction	0.03 ± 0.2	0.03	0.03 ± 0.2	0.03	–

Italic values indicate statistical significance at p < 0.05

*SD* standard deviation

Of the participants, 18% had some degree of incontinence (CCIS range 1–5) preoperatively, and 38% had some degree of incontinence after follow up (CCIS range 1–4). In the majority, there was no change in the degree of incontinence (n = 22, 65%) while 9 patients (27%) had worsening of incontinence and 3 patients (9%) had an improvement.

**Fig. 2:**
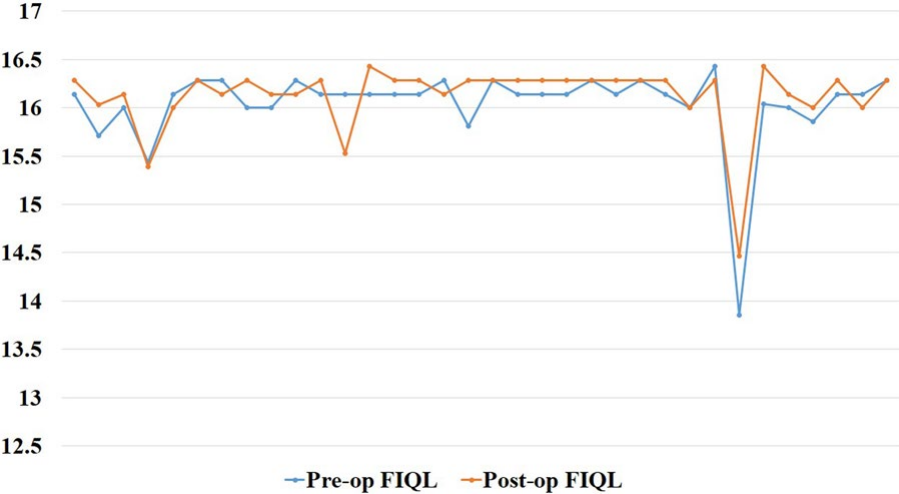
Figure plotting pre and post-op FIQL scores of individual patients

The mean overall FIQL calculated by adding the scores of 4 scales was 16.0 (SD ± 0.4) (95% confidence interval 15.9–16.2) preoperatively and 16.1 (SD ± 0.4) (95% confidence interval 16.0–16.2) postoperatively. There was no worsening of overall FIQL after surgery (Table 2). Plot showing pre and post-operative FIQL scores of individual patients is shown in Fig. 2.

**Table 2:** Comparison of mean preoperative and postoperative FIQL scores

	Pre-op FIQL ± SD	Standard error	Post-op FIQLI ± SD	Standard error
Total FIQL score (29)	16.0 ± 0.4	0.07	16.1 ± 0.4	0.06
Life style (10)	3.97 ± 0.1	0.02	3.98 ± 0.1	0.01
Coping behaviour (9)	3.98 ± 0.1	0.01	3.98 ± 0.1	0.01
Depression/self-perception (7)	4.14 ± 0.1	0.02	4.20 ± 0.2	0.03
Embarrassment (3)	3.95 ± 0.2	0.03	3.96 ± 0.2	0.03

*SD* standard deviation

There was no considerable difference in the pre and post-operative FIQL scores (15.9 vs 16.1 and 16.0 vs 16.2 respectively) in those with worsening incontinence compared to those without.

Greater difference was seen in scale measuring “depression/self-perception”. There was no difference in the mean score on “lifestyle” which measures the impact of incontinence on day to day activities and “embarrassment”.

Considering individual scores, the majority (n = 20, 59%) had an improvement in FIQL and 6 patients (18%) had no change and 8 patients (24%) had reduced FIQL. However, only 1 patient (3%) had reduction in scale “lifestyle” which measures the impact of incontinence on day-to-day activities.

## Discussion

This study has shown a significant increase in the mean FIQL scores following surgery for simple fistula-in-ano. Studies have been done to compare rates of incontinence and FIQL between simple and complex fistulae [12]. However, studies that prospectively assessed the degree of incontinence in simple fistulae before and after treatment are scant [4, 13].

The quality of life was mainly affected due to depression and self-perception. Furthermore, the difference seen in the scores measuring lifestyle was small. This was consistent with a similar study which showed that scores measuring psychological and social outcomes significantly improved following successful operative treatment of fistula-in-ano [11]. Therefore, it may be ideal to incorporate the intervention of a psychologist to improve the quality of life.

There was a statistically significant increase in the mean score measuring incontinence. However, the majority had no change in the degree of incontinence while 27% had mild worsening and 9% had an improvement. This is consistent with findings in a similar study which has reported worsening of incontinence in patients following surgery for simple fistulae [14] however, in the present study the degree of incontinence following surgery was low. This study also confirms that simple fistulae are also at risk of incontinence following surgery.

A cross-sectional study by Owen et al. [6] compared the quality of life with anal incontinence, using St. Marks incontinence score, showed a very low median score of 0 and there was no difference in the degree of incontinence in relation to recurrence of fistulae. Furthermore, in that study there was a significant reduction in the quality of life score compared to the normal population scores [6]. Thus in that study, although the scoring of degree of incontinence is low, there was a reduction in the quality of life of patients.

It was interesting to note that despite the increase in degree of incontinence, there was no worsening of quality of life related to anal incontinence. The reason may be that the statistically significant increase in the degree of incontinence was not clinically significant and did not have a significant impact on their day to day activities. That is probably due to the fact that, all the patients had only minor incontinence and had low mean incontinence scores both preoperatively and after follow up. The fact that there was no reduction in the faecal incontinence quality of life after surgery is important in terms of the outcome of the surgery. This indicates that those with low pre-operative incontinence scores in our cohort had not experienced worsening of faecal incontinence related quality of life, which is a major concern and a challenge for the surgeon.

Certain limitations in this study should be taken into consideration when interpreting the results. The observed increase in the quality of life may be influenced by the low degree of pre-operative and post-operative incontinence. Therefore the patient characteristics in our sample should be considered when interpreting the findings which were low pre-operative incontinence score and the presence of simple cryptoglandular fistula-in-ano.

Therefore, operative treatment could be offered despite the risk of worsening of anal incontinence in patients diagnosed to have simple cryptoglandular fistula-in-ano with low pre-operative incontinence scores as there was no worsening of quality of life in terms of anal incontinence after surgery.

## Conclusions

In this study, analysis of a cohort of simple cryptoglandular fistula-in-ano with low pre-operative incontinence showed no worsening in the FIQL following successful treatment despite minor worsening of incontinence. Since greater improvement was noted in scale measuring depression/self-perception, psychological interventions may be helpful before surgery to improve quality of life. We recommend large scale prospective studies to gain further insight on the impact of operative treatment on quality of life related to anal incontinence and to identify the contributory factors.

## Abbreviations

FIQL: faecal incontinence quality of life
CCIS: Cleveland clinic incontinence score
SD: standard deviation
TB: tuberculosis
QOL: quality of life
GIQLI: gastrointestinal quality of life index
SPSS: Statistical Package for the Social Sciences
